# Association Between Diabetic Foot Lesions and Diabetic Foot Ulcers: A Cross-Sectional Study

**DOI:** 10.3390/jcm15103754

**Published:** 2026-05-13

**Authors:** Moe Murai, Yoshitaka Hashimoto, Haruka Utsuyama, Takashi Ogasawara, Akifumi Shiota, Nozomi Yoshioka, Yusuke Hamazawa, Michiaki Fukui

**Affiliations:** 1 Department of Diabetes and Endocrinology, Matsushita Memorial Hospital, 5-55 Sotojima-cho, Moriguchi 570-8540, Japan; moe-h10@koto.kpu-m.ac.jp (M.M.);; 2 Department of Endocrinology and Metabolism, Graduate School of Medical Science, Kyoto Prefectural University of Medicine, Kyoto 602-8566, Japan

**Keywords:** diabetic foot ulcers, diabetic foot lesions

## Abstract

**Aims:** The purpose of this study was to clarify the prevalence of diabetic foot lesions and their association with lower-limb amputations and/or foot ulcers. **Materials and Methods:** In this cross-sectional study, 968 patients with diabetes were surveyed. Diabetic foot lesions were defined according to the broad national criteria, which encompass both mild abnormalities and more advanced conditions. Based on this definition, foot lesions were assessed using a questionnaire comprising the following 10 items: a history of lower-limb amputations, a history of treatment of foot lesions, numbness/loss of sensation/pain, skin discolorations, skin symptoms, nail abnormalities, foot ulcers/gangrene, foot deformities, foot infection, and intermittent claudication. Logistic regression analysis was used to examine risk factors for foot lesions. Area under the curve (AUC) of the number of foot lesions for lower-limb amputations or foot ulcers/gangrene was calculated using the receiver operating characteristic curve (ROC) analysis. **Results:** Approximately two-thirds of the patients had at least one type of diabetic foot lesion. Logistic regression analysis revealed that women, past or current smoking, a history of cardiovascular disease, and nephropathy were associated with the risk of diabetic foot lesions. According to the ROC analysis, the optimal cut-off point of number of diabetic foot lesions was three for identifying patients with a history of lower-limb amputation and/or the presence of foot ulcers or gangrene (AUC 0.80 (95% CI, 0.70–0.91), *p* < 0.01). **Conclusions:** Diabetic foot lesions are common in patients with diabetes and the prevalence of diabetic foot lesions was higher in patients with a history of lower-limb amputations and/or the presence of foot ulcers or gangrene. Early detection and care of diabetic foot lesions are necessary to prevent lower-limb amputations and foot ulcers.

## 1. Introduction

The prevalence of diabetes is increasing worldwide, with approximately 540 million adults currently affected [1]. The trend is also evident in Japan, where around 9 million patients are living with the disease [2].

Diabetic foot ulcers are an important diabetic complication. Approximately 20% of patients with diabetic foot ulcers complicated by moderate or severe infection undergo lower-limb amputation, primarily due to infection or progressive gangrene. In addition, the five-year mortality rate for patients with diabetic foot ulcers is approximately 30%. Furthermore, it has been reported that this rate exceeds 70% among patients who have undergone above-knee amputation [3]. It is reported that the prevalence of foot ulcers in patients with diabetes is 6.3% worldwide [4]. The regions with the highest prevalence are North America (13.0%), Africa (7.2%), Asia (5.5%), Europe (5.1%), and Oceania (3.0%) [4]. In contrast, the prevalence of foot ulcers among patients with diabetes in Japan is reported to be 0.7% [5]. Since these results are estimates based on a questionnaire survey, they may be lower than the global prevalence rate.

The etiology of diabetic foot ulcers is multi-factorial, involving a combination of mechanical pathways, such as diabetic sensory, motor, and autonomic neuropathy, and vascular pathways [3]. Loss of protective sensation due to sensory neuropathy, foot deformities, and biomechanical abnormalities due to motor neuropathy or direct mechanical damage and viscoelastic changes in the skin by autonomic neuropathy cause diabetic foot ulcers [3]. Furthermore, it has been reported that vascular complications in patients with diabetes progress through a series of processes involving multiple mechanisms, such as endothelial dysfunction, hypercoagulability, impaired angiogenesis, and intimal calcification [6].

In patients with diabetes, pre-ulcerative foot lesions frequently occur even in the absence of active ulcers. A Japanese cohort study found callus formation in 15–20% of patients, while nail and skin lesions were present in approximately 10–20% [7]. Cohort studies in Canada and China reported foot deformities in approximately 30–45% of patients [8,9]. The primary risk factors consistently preceding ulcer development include peripheral neuropathy, peripheral arterial disease, foot deformities, and a history of previous ulcers [8,10,11]. Early detection of diabetic foot lesions could play a key role in preventing diabetic foot ulcers and lower-limb amputations. However, studies reporting the prevalence of diabetic foot lesions, including mild cases, and their association with diabetic foot ulcers are extremely limited. Therefore, the present cross-sectional study aimed to investigate the prevalence of diabetic foot lesions and their association with diabetic foot ulcers and lower-limb amputations.

## 2. Materials and Methods

### 2.1. Study Design and Patients

For this study, we included 1040 patients with diabetes who visited the Department of Diabetes and Endocrinology, Matsushita Memorial Hospital, Moriguchi, Osaka, Japan, between 1 April and 31 July 2024. We excluded 72 patients (6.9%); thus, a total of 968 subjects, 590 men and 378 women, with Type 1 or Type 2 diabetes, were finally included in the analysis (Supplementary Figure S1). Approval for this study was granted by the Ethics Committee of Matsushita Memorial Hospital (approval number: 24030) and this study was conducted in accordance with the ethical standards of the 1964 Declaration of Helsinki and its later amendments. The study adopted an opt-out approach. Details were available within the hospital and on our website, and patients who did not consent to the use of their information were requested to contact us.

### 2.2. Definition of Diabetic Foot Lesions

In the Definition and Criteria for Diabetes-Related Foot Disease [12], foot lesion is defined as any abnormality associated with damage to the skin, nails, or deep tissues of the foot including both foot ulcers and pre-ulcerative lesions. A pre-ulcerative lesion is defined as a foot lesion that has a high risk of developing into a foot ulcer, such as an intra- or subcutaneous hemorrhage, blister, or skin fissure not penetrating into the dermis in a person at risk, whereas peripheral neuropathy, peripheral arterial disease, and foot deformities are considered diabetes-related foot risk factors for foot ulcer rather than lesions themselves. Although foot lesions include nail abnormalities in the broad definition of “foot disease,” nail changes are not specifically listed as pre-ulcerative lesions with high risk of ulceration. In contrast, the Japanese Society for Foot Care and Podiatric Medicine defines diabetic foot lesions as mild disorders—such as dry skin, cracks, and calluses due to peripheral neuropathy—to ulcers and foot deformities [13]. In this study, we adopted the latter definition of diabetic foot lesions, which includes mild lesions, in order to conduct an evaluation for early intervention. Foot lesions were evaluated using a questionnaire based on the following 10 items: (1) a history of lower-limb amputations, (2) a history of treatment of foot lesions, (3) numbness, loss of sensation or pain, (4) skin discolorations (such as erythema or bruising), (5) skin symptoms (including dry skin, cracks, and/or calluses), (6) nail abnormalities (including dyschromia, ingrown, or thickened nails), (7) foot ulcers and/or gangrene, (8) foot deformities (including hammertoe, mallet toe, bunions, metatarsal head protrusion, flat feet, etc.), (9) foot infections (including onychomycosis and/or cellulitis), and (10) intermittent claudication. In this study, the presence of at least one of the 10 items was defined as having diabetic foot lesions.

### 2.3. Data Collection

Data on age, sex, height, body weight, type of diabetes, age at onset of diabetes, duration of diabetes, exercise status, a history of drinking (none/social/almost every day) and a history of smoking (never/past/current), a family history of diabetes, the presence of microvascular disease, the presence of cardiovascular disease (CVD), blood pressure, medication for diabetes, presence of hypertension or dyslipidemia, and the use of antiplatelet or anticoagulant medication were collected from electronic medical records. Data on HbA1c, estimated glomerular filtration rate (eGFR), and urine albumin excretion were also obtained from electronic medical records. Body mass index (BMI) was assessed as body weight (kg) divided by height^2^ (m^2^). Exercisers were defined as those who reported engaging in physical activity outside of their daily routine, as ascertained by the questionnaire. A history of smoking was defined as never, past, and current smoking. The evaluation of diabetic retinopathy was conducted by ophthalmologists, who classified the condition as no- (NDR), simple- (SDR), pre-proliferative- (PPDR), or proliferative diabetic retinopathy (PDR) according to the International Clinical Diabetic Retinopathy Disease Severity Scale [14]. The nephropathy diagnosis was made on the basis of urine albumin excretion and eGFR according to KDIGO 2024 Clinical Practice Guideline for the Evaluation and Management of Chronic Kidney Disease [15]. In this study, the patients were evaluated as having neuropathy if they had complaints of sensory disturbance. Diabetes medications were identified as sulfonylureas, glinides, glucagon-like peptide-1 receptor agonists (GLP-1RA), dipeptidyl peptidase-4 inhibitors (DPP4is), thiazolidinediones, biguanides, imeglimins, sodium-glucose cotransporter-2 inhibitors (SGLT2is), α-glucosidase inhibitors (αGIs), and insulin. Hypertension was defined as either the use of antihypertensive medications or a blood pressure reading of 140/90 mmHg or higher. Dyslipidemia was defined according to any of the following criteria: taking dyslipidemia medications; having high-density lipoprotein cholesterol levels of less than 40 mg/dL; having low-density lipoprotein cholesterol levels of 140 mg/dL or higher; or having triglyceride levels of 150 mg/dL (fasting blood) or 175 mg/dL (anytime blood) or higher. A history of CVD was defined as stroke or ischemic heart disease.

### 2.4. Statistical Analysis

Statistical analysis was performed using EZR version 1.61 (Saitama Medical Center, Saitama, Japan) [16], which is a graphical user interface for R version 4.3.1, and R Commander version 2.8-0 designed to add statistical functions. Continuous variables were shown as median (interquartile range) and categorized variables as number (%). To assess differences between groups, statistical analyses such as the Mann–Whitney U test and Fisher’s exact test were employed. A *p* value lower than 0.05 was considered statistically significant. A logistic regression analysis was used to examine the risk factors for the presence of diabetic foot lesions. We chose age, BMI, sex, duration of diabetes equal to over 10 years, past or current smoker, exercise, a history of CVD, hypertension, dyslipidemia, insulin usage, retinopathy of PPDR or PDR, nephropathy of stage 2 or higher, alcohol, and type of diabetes as covariates. Of these, age, BMI, gender, duration of diabetes, a history of smoking, a history of CVD, hypertension, insulin use, and microvascular disease have been previously reported as risk factors for foot lesions, including diabetic ulcers [4,10,17]. It has also been reported that excessive alcohol consumption is a significant risk factor for lower-limb amputations and that moderate exercise decreases this risk [18].

Furthermore, receiver operating characteristic (ROC) curve analysis was used to calculate the area under the curve (AUC) for the number of diabetic foot lesions (excluding a history of lower-limb amputations and/or the presence of foot ulcers or gangrene; up to 8 items) in predicting having a history of lower-limb amputations and/or the presence of foot ulcers or gangrene. The optimal cut-off value was then determined.

## 3. Results

Supplementary Table S1 shows the characteristics of all patients in the study. Of the 968 patients enrolled, 590 (60.9%) were men, and 859 (88.7%) had Type 2 diabetes. The median age, duration of diabetes, BMI, and HbA1c levels were 68 [58–76] years, 13 [6–22] years, 24.2 [21.7–27.5] kg/m^2^, and 7.4 [6.8–8.2] %, respectively.

**Figure 1:**
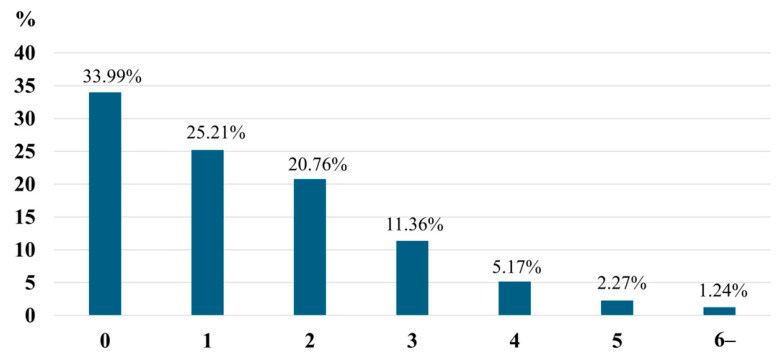
Prevalence of diabetic foot lesions by number of factors.

The results of the survey on diabetic foot lesions are shown in Table 1. The three most common lesions were nail abnormalities, sensory symptoms, and skin symptoms (Table 1). Foot ulcers/gangrene (1.24%) and a history of lower-limb amputations (0.83%) were also observed. The number of diabetic foot lesions is shown in Figure 1. The study found that only 34% had no diabetic foot lesions, 25.2% had one, and 40.8% had two or more. A total of 1.2% of them had six or more diabetic foot lesions.

The following 10 items were included in the diabetic foot lesions: (1) a history of lower-limb amputations, (2) a history of treatment of foot lesions, (3) numbness, loss of sensation or pain, (4) skin discolorations, (5) skin symptoms (including dry skin, cracks, and/or calluses), (6) nail abnormalities (including dyschromia, ingrown, or thickened nails), (7) foot ulcers and/or gangrene, (8) foot deformities, (9) foot infections (including onychomycosis and/or cellulitis), and (10) intermittent claudication.

Demographic and clinical characteristics of the participants according to the presence of diabetic foot lesions are shown in Table 2. The patients with diabetic foot lesions had a higher mean age, longer duration of diabetes, higher prevalence of microvascular disease and CVD, and higher urine albumin excretion compared to those without. The prevalence of hypertension and dyslipidemia, a history of CVD, and usage of antiplatelet drugs was significantly higher in the patients with diabetic foot lesions.

Table 3 shows the results of the risk factors for the presence of diabetic foot lesions. The analysis revealed that women (odds ratio [OR], 1.46; 95% confidence interval [CI], 1.04–2.06), past or current smokers (OR, 1.42; 95% CI, 1.02–1.98), a history of CVD (OR, 1.94; 95% CI, 1.22–3.09), and nephropathy (OR, 1.50; 95% CI, 1.09–2.05) were associated with the presence of diabetic foot lesions.

Multivariable logistic regression analysis was used to examine the risk factors for the presence of diabetic foot lesions. A history of cardiovascular diseases (CVDs) was defined as stroke or ischemic heart disease. Men were used as references for women. Non-smokers were used as references for past or current smokers. Non-exercise was used as a reference for exercise. Retinopathy was defined as having pre-proliferative diabetic retinopathy or proliferative diabetic retinopathy and no diabetic retinopathy or simple diabetic retinopathy was used as a reference. Nephropathy was defined as stage 2 or higher and stage 1 was used as a reference. Alcohol was defined as almost everyday alcohol consumption and no alcohol consumption or social alcohol consumption were used as references. Type 1 diabetes was used as a reference for Type 2 diabetes.

**Figure 2:**
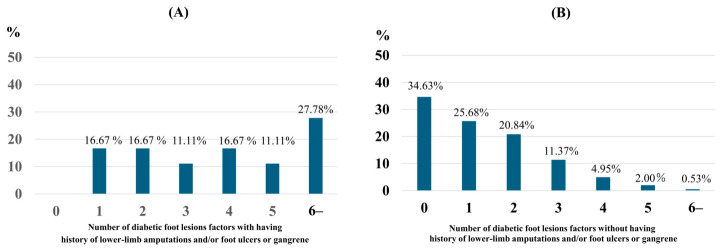
Prevalence of diabetic foot lesions by number of factors with or without a history of lower-limb amputations and/or the presence of foot ulcers or gangrene. (**A**) Among the patients with a history of lower-limb amputations and/or the presence of foot ulcers or gangrene. (**B**) Among the patients without a history of lower-limb amputations and/or the presence of foot ulcers or gangrene. Of 10 diabetic foot lesions, 8 items were used, excluding 2 items: a history of lower-limb amputations and the presence of foot ulcers or gangrene.

As shown in Figure 2A, individuals with a history of lower-limb amputations and/or the presence of foot ulcers or gangrene had at least one foot lesion, and nearly 30% had six or more lesions. Conversely, Figure 2B demonstrates that in individuals without such complications, the majority had few or no lesions, 35% had none, 25% had one, and 20% had two, while higher lesion counts were rarely observed.

The median number of diabetic foot lesions in the group with a history of lower-limb amputations and/or the presence of foot ulcers or gangrene was four (95%CI, 2–5.75), which was significantly higher than the median of one (95% CI, 0–2) in the group without these conditions (*p* < 0.001). According to the results of the ROC curve analysis, the optimal cut-off value for a history of lower-limb amputations and/or the presence of foot ulcers or gangrene in terms of the number of diabetic foot lesions (excluding a history of lower-limb amputations and the presence of foot ulcers or gangrene) was determined to be three (AUC 0.80 (95% CI 0.70–0.91), specificity = 0.904, sensitivity = 0.556, *p* < 0.01) (Supplementary Figure S2).

Patients with a history of lower-limb amputations and/or the presence of foot ulcers or gangrene had a significantly higher prevalence of all eight non-ulcer lesions than those without (Supplementary Table S2). Moreover, although the overall prevalence of a history of treatment of foot lesions (27.8% vs. 3.58%), skin discolorations (38.9% vs. 4.95%), and foot deformities (38.9% vs. 6.74%) was relatively low, individuals with a history of lower-limb amputations and/or the presence of foot ulcers or gangrene had significantly higher rates than patients without.

## 4. Discussion

This study revealed that 66% of patients with diabetes exhibited some form of diabetic foot lesion. Women, smokers, a history of CVD, and nephropathy were significantly related to the presence of diabetic foot lesions. This study also revealed a higher number of diabetic foot lesions in individuals with a history of lower-limb amputations and/or the presence of foot ulcers or gangrene and that having three or more diabetic foot lesions was the cut-off for having a history of lower-limb amputations and/or the presence of foot ulcers or gangrene.

Patients with diabetic foot ulcers have previously been reported to have significantly increased risk of all-cause mortality, fatal myocardial infarction, and cerebral infarction [17]. Therefore, early detection of diabetic foot lesions is important. As noted earlier, diabetic foot ulcers develop as a result of foot deformities and biomechanical abnormalities due to motor neuropathy, viscoelastic changes such as dry skin due to autonomic neuropathy, and sensory neuropathy [3]. In fact, as shown in Figure 2 and Supplementary Figure S2, a history of lower-limb amputations and/or the presence of foot ulcers or gangrene was associated with a higher prevalence of foot lesions, with a cut-off value of 3 representing these conditions. The high prevalence of sensory neuropathy and skin symptoms observed in the present study is consistent with previous reports identifying sensory neuropathy as one of the major risk factors for diabetic foot ulcers [3]. Furthermore, when the analysis was limited to patients with a history of lower-limb amputations and the presence of foot ulcers or gangrene, nail abnormalities were found to have a high prevalence. In patients with diabetes, factors such as peripheral neuropathy, peripheral artery disease, and an increased susceptibility to fungal infections often contribute to these nail abnormalities [19]. Previous studies have also reported that calluses, onychomycosis, and ingrown nails themselves are pre-ulcer lesions that lead to foot ulcer formation, causing foot inflammation and pain [7]. This study considers diabetic foot lesions as a progressive continuum, advancing from mild skin and structural abnormalities to foot ulcers and lower-limb amputations, positioning foot ulcers as a critical turning point in progression toward severe complications. Foot ulcers are highly likely to develop as a result of the accumulation of preceding foot lesions. In this context, our ROC analysis identified a cut-off of three or more lesions (AUC = 0.80), suggesting that the risk of ulceration increases particularly when multiple lesions accumulate. This finding reinforces the concept that early, seemingly mild lesions are not isolated findings but components of a trajectory that may culminate in a diabetic foot ulcer. Therefore, during the examination of patients with diabetes, it is desirable to conduct detailed foot examinations within an appropriate scope to ensure early detection of these mild foot lesions and prompt therapeutic intervention.

The present study identified several risk factors for diabetic foot lesions, including female sex, smoking, a history of CVD, and nephropathy. A multitude of risk factors for diabetic foot ulcers have been identified in previous reviews. These include older age, male sex, Type 2 diabetes, poor glycemic control, duration of diabetes, low BMI, hypertension, microvascular complications, usage of insulin, and a history of smoking [4,10]. In addition, the reported prevalence of foot lesions other than foot ulcers was about 25% with diabetic neuropathy, 12% with peripheral arterial disease (PAD), and 30% with foot deformities [8]. The following risk factors have been identified as contributing to the development of foot deformities: female sex, age greater than 40 years, hypertension, and hyperlipidemia. The prevalence of foot deformities increases with age, with a higher incidence observed among women compared to men. Some individuals attribute this higher prevalence to factors such as walking patterns and the use of inappropriate footwear [8]. In the present study, the prevalence of foot deformities was 7.33% overall, reaching 38.9% in patients with a history of lower-limb amputations and/or the presence of foot ulcers or gangrene, which is consistent with previous reports indicating that the prevalence of foot deformities is higher than that of PAD [8]. In addition, based on this previous report [8], the inclusion of foot deformities and calluses in the definition of diabetic foot lesions may have contributed to the finding that female sex was associated with the development of diabetic foot lesions in this study. Early detection of foot deformities is therefore essential for preventing foot ulcer formation. In fact, it has been established that foot deformities lead to changes in plantar pressure and an increased incidence of foot ulcers [11]. In the Prevalence and Risk Assessment of Foot Lesions in Type 1 and Type 2 Diabetes in a Large Canadian Population (PEDAL Study), loss of sensation, skin symptoms such as hyperkeratosis, and onychomycosis were each significantly associated with ulcer formation [9]. This study also found that there were significantly more diabetic foot lesions in patients with a history of lower-limb amputations and/or the presence of foot ulcers or gangrene. Consistent with previous reports, the present study found a relatively high prevalence (66%) of diabetic foot lesions—including onychomycosis, loss of sensation, foot deformities, and calluses—which may contribute to foot ulcer formation.

Several limitations of this study should be acknowledged. First, this was a self-administered survey, and there is a possibility of omissions or errors. Second, the cross-sectional study design limits the ability to ascertain causal relationships, including causal links between diabetic foot lesions and ulceration or amputation. Third, we were unable to adjust for potential confounding factors that could influence the prevalence of diabetic foot lesions or ulcer risk, such as the duration of glycemic control, footwear habits, type of physical activity, the presence of PAD, and severity of neuropathy. Fourth, although the questionnaire comprehensively covers common diabetic foot lesions, we cannot deny the possibility of substantial reporting bias. Fifth, stratified analysis by lesion type could provide more meaningful insights. Unfortunately, however, clinical severity, location, and depth of lesions, which are critical factors influencing ulcer risk, were not assessed in this study and thus, we could not perform stratified analysis. Sixth, the type of amputation and whether diabetic lesions were located on the same or contralateral limb are important pieces of information. Unfortunately, these data were not collected in the present study. Seventh, the broad definition of diabetic foot lesions adopted in this study, which encompasses 10 heterogeneous items ranging from mild abnormalities (such as nail dyschromia or dry skin) to advanced conditions (such as foot ulcers and gangrene), might limit the robustness of our conclusions. Although a more detailed analysis would have been desirable, this was not feasible due to the questionnaire-based survey design and the limited number of serious adverse events. This methodological heterogeneity is also reflected in the low sensitivity (0.556) of the cut-off value of “three lesions,” which was derived from the ROC curve for predicting lower-limb amputation and/or foot ulcers/gangrene. Although the AUC was 0.80, the low sensitivity suggests that simply counting the number of lesions may be insufficient as a screening tool, and future studies will require a more refined definition that incorporates lesion severity. Eighth, in this study, because a questionnaire was used to assess the presence or absence of sensory neuropathy, validated assessment scales such as the Neuropathy Disability Score (NDS) and the Michigan Neuropathy Screening Instrument (MNSI) were not employed; furthermore, the study did not include an objective assessment of loss of protective sensation (LOPS), which is identified as a major risk factor for the development of foot ulcers in the 2023 IWGDF guidelines. Ninth, the single-center design and the use of national criteria (those of the Japanese Society for Foot Care and Podiatric Medicine) for defining diabetic foot lesions may limit the reproducibility of our findings. Lastly, because this study included only Japanese participants, it remains uncertain whether the findings can be generalized to other racial or ethnic groups. Cultural and environmental factors, such as differences in ulcer-prone sites related to varying shoe wearing habits across countries, including Japan’s custom of removing shoes indoors, as well as disparities in healthcare access and the prevalence of peripheral arterial disease, may influence both the distribution of foot lesions and ulcer risk. Further studies in other countries are needed to clarify the prevalence of foot lesions and to determine whether the relationship between foot lesions and ulcer development observed in this study holds true in different populations.

## 5. Conclusions

The prevalence of any type of diabetic foot lesion was approximately 66%, including minor lesions in addition to foot ulcers. A history of lower-limb amputations and/or the presence of foot ulcers or gangrene was associated with a higher prevalence of foot lesions, with a cut-off value of 3 representing a history of lower-limb amputations and/or the presence of foot ulcers or gangrene. Although early identification and management of diabetic foot lesions may play an important role in preventing foot ulcers, gangrene, and lower-limb amputations, the cross-sectional nature of this study limits our ability to determine causal links between these lesions and subsequent ulceration or amputation.

## Figures and Tables

**Table 1:** Details of the presence or absence of diabetic foot lesions.

Total *n* = 968	
A history of lower-limb amputations	8 (0.83%)
A history of treatment of foot lesions	39 (4.02%)
Numbness, loss of sensation, or pain	288 (29.75%)
Skin discolorations	54 (5.58%)
Skin symptoms (including dry skin, cracks, or calluses)	234 (24.17%)
Nail abnormalities (including dyschromia, ingrown, or thickened nails)	366 (37.81%)
Foot ulcers or gangrene	12 (1.24%)
Foot deformities	71 (7.33%)
Foot infections (including onychomycosis and cellulitis)	190 (19.63%)
Intermittent claudication	110 (11.36%)

Expressed as a number (%).

**Table 2:** Demographic and clinical characteristics according to the presence of diabetic foot lesions.

	Diabetic Foot Lesions (+) (*n* = 639)	Diabetic Foot Lesions (−) (*n* = 329)	*p*
Sex (men/women)	376/263	214/115	0.071
Age (years)	70.0 (59–77)	65(55–75)	<0.001
Duration of diabetes (years)	14 (6–24)	12 (6–21)	0.034
Duration of diabetes ≥ 10 years (−/+)	226/412	132/196	0.159
Type of diabetes (Type 1/Type 2)	65/574	44/285	0.163
HbA1c (%)	7.4 (6.8–8.2)	7.3 (6.7–8.2)	0.095
Body mass index (kg/m^2^)	24.2 (21.7–27.7)	24.2 (21.5–27.2)	0.482
Retinopathy (NDR/SDR/PPDR/PDR) (*n* = 894)	406/104/21/65	223/54/4/17	0.012
Nephropathy (stage 1/2/3/4/5) (*n* = 961)	307/211/78/32/9	200/84/31/8/1	0.001
eGFR (mL/min/1.73 m^2^) (*n* = 967)	70.0 (51.6–84.4)	71.5 (59.2–87.1)	0.0308
Urine albumin excretion (mg/gCr) (*n* = 954)	30.9 (11.8–128.8)	18.5 (8.9–80.2)	<0.001
A family history of diabetes (+/−) (*n* = 631)	216/198	100/117	0.155
A history of CVD (+/−)	125/514	34/295	<0.001
Exercise status (+/−) (*n* = 947)	142/484	87/234	0.149
Systolic blood pressure (mmHg) (*n* = 953)	131 (122–141)	130 (121–140.5)	0.512
Smoking (never/past or current) (*n* = 963)	289/347	166/161	0.118
Alcohol (none/social/almost every day) (*n* = 959)	355/154/122	188/78/62	0.956
DPP4i (+/−)	355/304	182/147	0.415
GLP-1RA (+/−)	141/498	56/273	0.077
SGLT2i (+/−)	313/326	151/178	0.378
Other oral hypoglycemic agents (+/−)	423/221	216/108	0.774
Insulin (+/−)	240/399	112/217	0.291
Hypertension (+/−)	364/275	161/168	0.021
Antihypertensive medication (+/−)	353/286	156/173	0.025
Dyslipidemia (+/−)	359/280	158/171	0.017
Antiplatelet drug (+/−)	126/513	46/283	0.027
Anticoagulant drug (+/−)	28/611	13/316	0.867

Abbreviation: NDR, no diabetic retinopathy; SDR, simple diabetic retinopathy; PPDR, pre-proliferative diabetic retinopathy; PDR, proliferative diabetic retinopathy; eGFR, estimated glomerular filtration rate; CVD, cardiovascular diseases; DPP4i, dipeptidyl peptidase-4 inhibitors; GLP-1RA, glucagon-like peptide-1 receptor agonists; SGLT2i, sodium glucose co-transporters 2 inhibitors. Other oral hypoglycemic agents include sulfonylurea, glinide, biguanide, thiazolidine, imeglimin, and alpha-glucosidase inhibitors. Categorized variables were expressed as number and difference was evaluated by chi-square test. Continuous variables were expressed as medians (25th–75th percentiles) and difference was evaluated by Mann–Whitney U test.

**Table 3:** Risk factors for the presence of diabetic foot lesions.

Factors	Odds Ratio (95% CI)	*p* Value
Age, years	1.01 (1.00–1.02)	0.053
Body mass index, kg/m^2^	1.00 (0.99–1.01)	0.592
Women	1.46 (1.04–2.06)	0.030
Duration of diabetes ≥ 10 years	0.81 (0.57–1.14)	0.227
Past or current smoker	1.42 (1.02–1.98)	0.037
Exercise	0.80 (0.57–1.12)	0.202
A history of CVD	1.94 (1.22–3.09)	0.005
Hypertension	0.94 (0.68–1.30)	0.715
Dyslipidemia	1.11 (0.82–1.52)	0.493
Insulin usage	1.27 (0.89–1.83)	0.191
Retinopathy	1.64 (0.95–2.83)	0.075
Nephropathy	1.50 (1.09–2.05)	0.013
Alcohol consumption	1.10 (0.75–1.62)	0.614
Type 2 diabetes	1.28 (0.76–2.14)	0.354

## Data Availability

Data from this study are available from the corresponding author upon reasonable request.

## Supplementary Materials

The following supporting information can be downloaded at: https://www.mdpi.com/article/10.3390/jcm15103754/s1, Table S1: Demographic and clinical characteristics of the overall participants; Table S2: Relationship between a history of lower-limb amputations and/or the presence of foot ulcers or gangrene and the other diabetic foot lesions; Figure S1: Flow diagram illustrating the process for selecting the study population; Figure S2: Receiver operating characteristic (ROC) curve and area under the ROC curve (AUC) showing the ability of number of diabetic foot lesions to determine a history of lower-limb amputations and/or the presence of foot ulcers or gangrene.
