# Supplementary material for: Association Between Diabetic Foot Lesions and Diabetic Foot Ulcers: A Cross-Sectional Study
Source: J Clin Med. 2026 May 13;15(10):3754. doi: 10.3390/jcm15103754 (PMC13207091; doi:10.3390/jcm15103754)
Supplement: Supplementary file 1 [file jcm-15-03754-s001.zip › Supplementary Tables.pdf]

Supplementary Table S1. Demographic and clinical characteristics of the overall participants

	Total n = 968
Sex (Men/Women)	590 (61.0%)/378 (39.0%)
Age (years)	68(58—76)
Duration of diabetes (years) (n = 966)	13 (6—22)
Duration of diabetes ≥10 years (−/+) (n = 966)	358 (37.1%)/608 (62.9%)
Type of diabetes (Type 1/Type 2)	109 (11.3%)/859 (88.7%)
HbA1c (%)	7.4 (6.8—8.2)
Body mass index (kg/m^2) (n = 966)	24.2 (21.7—27.5)
Retinopathy (NDR/SDR/PPDR/PDR) (n = 894)	629 (70.4%)/158 (17.7%)/25 (2.8%)/82 (9.2%)
Nephropathy (stage 1/2/3/4/5) (n = 961)	507 (52.8%)/295 (30.7%)/109 (11.3%)/40 (4.2%)/10 (1.0%)
eGFR (ml/min/1.73 m^2) (n = 967)	70.3 (54—85.4)
Urine albumin excretion (n = 954)	26.2 (10.90—108.7)
A history of CVD (+/−)	159 (16.4%)/809 (83.6%)
Systolic blood pressure (n = 953)	131 (121—141)
Smoking (never/past or current) (n = 963)	455 (47.2%)/508 (52.8%)
Alcohol (none/social/almost every day) (n = 959)	543 (56.6%)/232 (24.2%)/184 (19.2%)
DPP4i (+/−)	517 (53.4%)/451 (46.6%)
GLP-1RA (+/−)	197 (20.4%)/771 (79.6%)

SGLT2i (+/−)	464 (47.9%)/504 (52.1%)
Other oral hypoglycemic agents (+/−)	644 (66.5%)/324 (33.5%)
Insulin (+/−)	352 (36.4%)/616 (63.6%)
Hypertension (+/−)	525 (54.2%)/443 (45.8%)
Antihypertensive medication (+/−)	509 (52.6%)/459 (47.4%)
Dyslipidemia (+/−)	517 (53.4%)/451(46.6%)
Antiplatelet drug (+/−)	171 (17.7%)/796 (82.2%)
Anticoagulant drug (+/−)	41 (4.2%)/927 (95.8%)

Abbreviation: NDR, no diabetic retinopathy; SDR, simple diabetic retinopathy; PPDR, pre-proliferative diabetic retinopathy; PDR, proliferative diabetic retinopathy; eGFR, estimated glomerular filtration rate; CVD, cardiovascular diseases; DPP4i, dipeptidyl peptidase-4 inhibitors; GLP-1RA, glucagon-like peptide-1 receptor agonists; SGLT2i, sodium glucose co-transporters 2 inhibitors.

Other oral hypoglycemic agents include sulfonylurea, glinide, biguanide, thiazolidine, imeglimin, and alpha-glucosidase inhibitors.

Data were expressed as number or medians (25th–75th percentiles).

Supplementary Table S2. Relationship between a history of lower-limb amputations and/or the presence of foot ulcers or gangrene and the other diabetic foot lesions

	A history of lower-limb amputations and/or the presence of foot ulcers or gangrene		p
	(+) *n* = 18	(−) *n* = 950	
A history of treatment of foot lesions (+/−)	5 (27.8%)/13 (72.2%)	34 (3.58%)/916 (96.4%)	<0.001
Numbness, loss of sensation, or pain (+/−)	13 (72.2%)/5 (27.8%)	275 (28.9%)/675 (71.1%)	<0.001
Skin discolorations (+/−)	7 (38.9%)/11 (61.1%)	47 (4.95%)/903 (95.1%)	<0.001
Skin symptoms (including dry skin, cracks, or calluses) (+/−)	11 (61.1%)/7 (38.9%)	223 (23.5%)/727 (76.5%)	<0.001
Nail abnormalities (including dyschromia, ingrown, or thickened nails) (+/−)	16 (88.9%)/2 (11.1%)	350 (36.8%)/600 (63.2%)	<0.001
Foot deformities (+/−)	7 (38.9%)/11 (61.1%)	64 (6.74%)/886 (93.3%)	<0.001
Foot infections (including onychomycosis and cellulitis) (+/−)	7 (38.9%)/11 (61.1%)	183 (19.3%)/767 (80.7%)	0.0641

Intermittent claudication (+/−)	5 (27.8%)/13 (72.2%)	105 (11.1%)/845 (88.9%)	0.0441

The differences were evaluated by chi-square test.

Supplemental Figure Captions

Supplementary Figure S1. Flow diagram illustrating the process for selecting the study population.

Supplementary Figure S2. Receiver operating characteristic (ROC) curve and area under the ROC curve (AUC) showing the ability of number of diabetic foot lesions to determine a history of lower-limb amputations and/or the presence of foot ulcers or gangrene

Of the 10 diabetic foot lesions, 8 items were used, excluding 2 items: A history of lower-limb amputations, the presence of foot ulcers or gangrene. According to the ROC analysis, the optimal cut-off point of number of diabetic foot lesions was 3 (AUC 0.80 (95% CI 0.70–0.91), specificity = 0.904, sensitivity = 0.556, $p < 0.01$).
