# Supplementary figures and images for: Association Between Diabetic Foot Lesions and Diabetic Foot Ulcers: A Cross-Sectional Study
Source: J Clin Med. 2026 May 13;15(10):3754. doi: 10.3390/jcm15103754 (PMC13207091; doi:10.3390/jcm15103754)

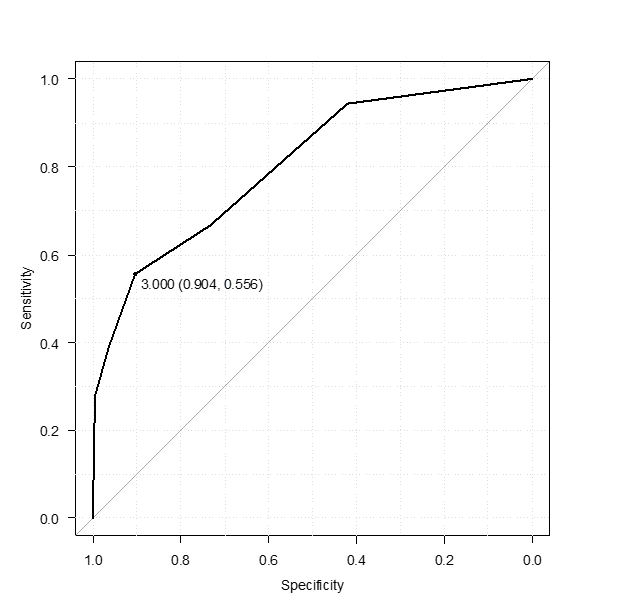

Supplement: Supplementary file 1 [file jcm-15-03754-s001.zip › Supplementary Figure S2.tif]
